# Exploring Perfluoroalkyl Substances (PFASs) in Aquatic Fauna of Lake Trasimeno (Italy): Insights from a Low-Anthropized Area

**DOI:** 10.3390/toxics12030196

**Published:** 2024-03-01

**Authors:** Tommaso Stecconi, Arianna Stramenga, Tamara Tavoloni, Simone Bacchiocchi, Martina Ciriaci, Francesco Griffoni, Paolo Palombo, Gianni Sagratini, Melania Siracusa, Arianna Piersanti

**Affiliations:** 1 School of Pharmacy, University of Camerino, 62032 Camerino, Italy; tommaso.stecconi@unicam.it (T.S.); gianni.sagratini@unicam.it (G.S.); 2 Istituto Zooprofilattico Sperimentale Dell’umbria e Delle Marche “Togo Rosati”, 60131 Ancona, Italy; a.stramenga@izsum.it (A.S.); s.bacchiocchi@izsum.it (S.B.); m.ciriaci@izsum.it (M.C.); f.griffoni@izsum.it (F.G.); p.palombo@izsum.it (P.P.); m.siracusa@izsum.it (M.S.); a.piersanti@izsum.it (A.P.)

**Keywords:** perfluorinated compounds, freshwater ecosystem, fishes, crustaceans, contamination profiles

## Abstract

This study investigated the concentrations and profiles of 19 perfluoroalkyl substances (PFASs) in the muscle and liver of four freshwater species from Lake Trasimeno (Italy): *Anguilla anguilla* (European eel), *Carassius auratus* (goldfish), *Perca fluviatilis* (European perch), and *Procambarus clarkii* (red swamp crayfish). In livers, the amount of PFASs ranged from 3.1 to 10 µg kg^−1^, significantly higher than that in muscle (0.032–1.7 µg kg^−1^). The predominant PFASs were perfluorooctane sulfonic acid (PFOS) and long-chain carboxylic acids (C8–C14). Short-chain compounds (C4–C5), as well as the long-chain sulfonic acids (C9–C12), were not quantified. The contamination patterns were similar among species with few differences, suggesting the influence of species-specific accumulation. The PFAS concentrations in livers were comparable among species, while in muscle, the higher values were measured in European eel, followed by goldfish, European perch, and red swamp crayfish. The levels were generally lower than those reported for fish from Northern Italian lakes and rivers. The concentrations of regulated PFASs were lower than the maximum limits set by Regulation EU 2023/915 and did not exceed the Environmental Quality Standards (PFOS in biota). This study provides the first valuable insights on PFASs in freshwater species from Lake Trasimeno.

## 1. Introduction

Perfluoroalkyl substances (PFASs) are a family of synthetic compounds characterized by carbon–fluorine bonds (C-F), with exceptional strength and stability, as well as hydrophobic and hydrophilic characteristics, rendering them valuable in a variety of industrial applications since the 1940s [1,2,3]. The widespread use of these hardly-degradable substances has led to their release into the environment through production processes, PFAS-containing product usage, and waste disposal. PFASs are widely detected in various environmental compartments, including water, sediments, soils, air, and biota, where they can persist for extended periods and bio-accumulate in the tissues of living organisms [4,5]. PFAS pollution is considered a global problem. Perfluorooctanoic acid (PFOA) and perfluorooctane sulfonate (PFOS), the most known and investigated PFASs, are involved in hepatotoxicity, developmental toxicity, and immunotoxicity inducing reproductive and hormonal effects, and they have been included in the list of dangerous substances for which restrictions on manufacturing and use have been set [6,7,8]. PFOS and its salts, PFOA and perfluorohexane sulfonic acid (PFHxS), were either listed, or recommended for listing, in the Stockholm Convention on Persistent Organic Pollutants (POPs) [9,10].

In July 2020, the European Food Safety Authority (EFSA) CONTAM Panel set a safety threshold for the perfluoroalkyl substances mainly contributing to the human serum levels: PFOA, PFOS, perfluorononanoic acid (PFNA), and PFHxS. A tolerable weekly intake (TWI) of 4.4 ng kg^−1^ of body weight (bw) per week was set for their sum [7].

As for many other organic contaminants, diet is the main route of human exposure to PFASs [6,7]. On 24 August 2022, the European Union issued Recommendation 2022/1431, asking member states to monitor the presence of the four abovementioned PFASs in food, along with other 18 similar compounds and 6 emerging PFASs, with the aim of assessing the background contamination levels [11]. On 1 January 2023, Commission Regulation (EU) 2022/2388 set maximum limits for PFOA, PFOS, PFNA, PFHxS, and their sum in food [12,13].

The ongoing monitoring of PFASs in various environmental compartments is of utmost importance; surely, aquatic ecosystems are of primary interest because they act as possible sinks, collecting toxic contaminants [5,14,15].

Of particular concern is the bioaccumulation of PFASs in aquatic organisms, occurring through complex ecological processes in the aquatic food web. The consumption of fish and fish products was identified as one of the main routes of human exposure, and even minimal levels of fish product consumption have been linked to elevated concentrations of PFASs in human populations [7].

Concern regarding the adverse effects of pollutants in aquatic environments is also reflected in Directive 2008/105/EU, which pertains to priority substances in water policy [16,17]. This directive amends the Water Framework Directive 2000/60/EC and establishes Environmental Quality Standards (EQSs) for specific persistent organic pollutants (POPs), including PFOS, in both surface water and biota, with the objective of safeguarding human health and the environment [18].

PFAS levels in the muscle of freshwater fishes from Northern European lakes (Sweden and Finland), Germany, and the Netherlands, are available [19,20,21,22,23]. Italian freshwater ecosystems have also been investigated for PFASs, especially those located in the north of the country. Giari et al. compared European eels caught in the Po River and in the Comacchio Lagoon and, in a further study, expanded the investigation to other fish species from the Po River, including bleak, channel catfish, and barbel [24,25]. Chiesa et al. monitored 16 PFASs in Garda Lake European eel and European perch, which are among the most consumed freshwater species [26,27]. PFASs were also studied in aquatic species from Varese Lake, Mergozzo Lake, and other subalpine Italian lakes [28,29,30,31]. All these studies investigated the presence and distribution of PFASs in various aquatic ecosystems, focusing on freshwater species from different geographical regions. The results revealed different profiles, concentrations, and PFAS patterns, characterized by the predominance of PFOS. Their ubiquitous presence, even in pristine ecosystems, highlights the need for comprehensive monitoring and risk assessment.

The primary goal of this study is to investigate the presence of PFASs in Lake Trasimeno, the largest in Central Italy, located in the Umbria region. Nineteen PFASs, including both carboxylic and sulphonic acid, as well as the branched isomers of PFHxS and PFOS, were analyzed in the muscle and liver tissues of fishes and crustaceans collected in 2021. Four species among the most widely consumed were selected for PFAS analysis: European eel (*Anguilla anguilla*), European perch (*Perca fluviatilis*), goldfish (*Carassius auratus*,), and red swamp crayfish (*Procambarus clarkii*). Lake Trasimeno is a natural basin, surrounded mainly by agricultural activities. Although previous research examined the presence of other contaminants, such as brominated flame retardants (BFRs), polychlorinated biphenyls (PCBs), heavy metals, and organochlorine pesticides, in the local fauna, the present study is the first dealing with PFASs [32,33]. The monitoring of freshwater species has a dual purpose: to ensure consumer health by assessing food safety, since Lake Trasimeno fishes are an important and renowned food resource for the local population, and to provide insight on the baseline contamination levels in a low-industrialized ecosystem by comparing the measured levels to the Environmental Quality Standards (EQSs).

## 2. Materials and Methods

### 2.1. Characterization of the Study Area

Lake Trasimeno (43°9′11″ N, 12°5′ E) is the largest laminar lake in Italy. It boasts a surface area of 126 km^2^ and an average and maximum depth of 4.72 m and 6.3 m, respectively. The lake is renowned for its abundant fish population, encompassing 19 different species, with the majority belonging to the Cyprinidae family [32,34]. Traditional fishing is a vital economic activity in the region, supported by governmental initiatives that promote the production and consumption of local food [34,35].

As part of the official monitoring program established in accordance with EC Regulation 2017/644, freshwater fish and crayfish samples were collected in 2021 by local fishermen. Four species, among those more widely consumed, were selected for PFAS analysis: European eel (*Anguilla anguilla*, n = 9), European perch (*Perca fluviatilis*, n = 16), goldfish (*Carassius auratus*, n = 7), and red swamp crayfish (*Procambarus clarkii*, n = 41). A total of 73 specimens were collected. Muscle and liver tissues of each fish were analyzed. In *P. clarkii*, only the abdomen was processed, after carapace and rostrum removal. The crayfish does not have a proper liver but instead has a hepatopancreas; these organs were not analyzed because it was not possible to collect the amounts of tissue necessary for the analysis. All the samples of European eel and goldfish were individually analyzed, while in the case of European perch and red swamp crayfish (since the size of the animals was too small to have the amount of material needed for the analysis), the specimens were pooled to form 8 and 9 laboratory samples respectively, as detailed in Table 1 (biometric data in Table S1). All collected samples were homogenized, frozen at −20 °C, and stored in the laboratory until PFAS analysis.

### 2.2. Standards and Reagents

Commercial solutions of native (PFAC-MXC) and labelled (MPFAC-C-ES) PFASs at 2 μg mL^−1^ in MeOH and of syringe standard ^13^C_4_- PFOS at 50 μg mL^−1^ in MeOH were purchased from Wellington Laboratories (Guelph, ON, Canada). Details regarding all the target compounds and internal standards are listed in Table 2. Methanol (MeOH), LC–MS acetonitrile (ACN), and ammonia solution 30% (NH_4_OH) were purchased from Honeywell (Charlotte, NC, USA). Ultrapure water was produced by a Milli-Q purification apparatus (Millipore, Bedford, MA, USA). Acetic acid (CH_3_COOH) and sodium acetate (CH_3_COONa) were obtained from VWR International Srl (Radnor, PA, USA), and n-Nonane and ammonium acetate (CH_3_COONH_4_) were obtained from Sigma-Aldrich (St. Louis, MO, USA). SPE polymeric weak anion exchange cartridges Strata X-AW (200 mg/6 mL) were obtained from Phenomenex (Torrance, CA, USA), and Supelclean™ ENVI-Carb™ SPE Bulk Packing (120/400 mesh) was obtained from Sigma-Aldrich (St. Louis, MO, USA). A certified reference material, IRMM-427 (PFASs in fish tissue), was supplied by the Joint Research Center (Geel, Belgium).

### 2.3. Sample Extraction and Analysis

The applied method, enabling the simultaneous analysis of 11 perfluoroalkyl carboxylic acids (PFCAs: PFBA, PFPeA, PFHxA, PFHpA, PFOA, PFNA, PFDA, PFUnDA, PFDoDA, PFTrDA, PFTeDA) and 8 perfluoroalkane sulfonic acids (PFSAs: PFBS, PFPeS, PFHxS, PFHpS, PFOS, PFNS, PFDS, PFDoDS), including the branched isomers of PFHxS (*br*-PFHxS) and PFOS (*br*-PFOS), has been previously described [36,37]. Briefly, 2 g of muscle and 1.0 g of liver were weighed in a 50 mL polypropylene centrifuge tube, spiked with 0.40 ng of the label internal standards, extracted twice with 10 mL of ACN, shaken (15 min), sonicated (15 min), and centrifuged (3220 rcf, 15 min, 2 °C). The organic phase was withdrawn and the volume reduced to 5 mL under nitrogen stream (≤40 °C). The extract was frozen at *−*20 °C overnight, then centrifuged (3220 rcf, 20 min, 2 °C), and the supernatant was diluted with 40 mL of water prior to SPE clean-up on STRATA X-AW columns (200 mg/6 mL). The sample was loaded onto the conditioned columns and eluted with 2% NH_4_OH in MeOH into a 15 mL polypropylene tube containing 80 mg of graphitized carbon activated with 100 μL of acetic acid (d-SPE). The final extract was brought to dryness and reconstituted with 200 μL of a ^13^C_4_–PFOS syringe standard at 2 ng mL^−1^.

LC–MS/MS analysis was conducted on an Acquity ultra-performance liquid chromatography (UPLC) system coupled with a Xevo TQ-S quadrupole mass spectrometer (Waters Corporation, Milford, MA, USA), operating in multiple reaction monitoring (MRM) mode. The instrumental conditions are given in Table S2 [36,37]. Chromatographic separation was achieved on a Luna Omega PS C18 column (100 mm × 2.1 mm, 1.6 μm, Phenomenex, Torrance, CA, USA), and a second chromatography on a Raptor C18 column (100 × 2.1 mm, 2.7 µm, Restek, Bellefonte, PA, USA) was employed to unequivocally distinguish the short-chain PFBA and PFPeA, for which only one transition was available for mass detection. Branched isomers (*br*-PFHxS and *br*-PFOS) were quantified by comparing the linear compounds’ response (*L*-PFHxS and *L*-PFOS) to the transition m/z 499 > 80 and 399 > 80, respectively.

### 2.4. Quality Assurance and Quality Control

The comprehensive method validation was previously discussed [37]. The achievable quantification limits (LOQs) for all the analytes were 0.010 µg mL^−1^, except for PFBA (0.20 µg mL^−1^). Each batch included the analysis of a procedural blank, a blank sample, the same blank sample spiked at 0.050 μg kg^−1^, and the certified reference material (CRM) IRMM-427 Pike-Perch. Background contamination was carefully monitored at every stage of the analytical process.

The results are reported in X-charts (recoveries), R-charts (repeatability), and background contamination charts. The recoveries of the labelled internal standards for each sample were also recorded.

Regular participation in the European Union Reference Laboratory (EURL) for Halogenated POPs proficiency tests (PTs) was guaranteed.

### 2.5. Data Analysis

PFAS concentrations below the LOQs were treated using the “lower bound” approach (concentrations < LOQ = 0). The PFAS sum (ΣPFASs) was calculated as a lower bound (l.b.) both as a sum of the nineteen PFASs (Σ19PFASs) and as a sum of the four regulated substances PFOA, PFOS, PFNA, and PFHxS (Σ4PFASs) [7,11,13].

Principal component analysis (PCA) was performed to investigate the variation in PFAS profiles. Individual PFASs are expressed as percentages of the 19 PFAS sum. All the statistical analyses were performed using the R-based software CAT (Chemiometric Agile Tool by R. Leardi, C. Melzi, G. Polotti, downloadable from http://gruppochemiometria.it/index.php/software; R Version 3.1.2 2014-10-31; accessed on 4 July 2023).

## 3. Results and Discussion

Nineteen linear-chain PFASs were analyzed, including eleven perfluoroalkyl carboxylic acids (PFCAs: PFBA, PFPeA, PFHxA, PFHpA, PFOA, PFNA, PFDA, PFUdA, PFDoA, PFTrDA, PFTeDA) and eight perfluoroalkane sulfonic acids (PFSAs: L-PFBS, L-PFPeS, PFHxS, L-PFHpS, PFOS, L-PFNS, L-PFDS, L-PFDoS). The total PFOS (tot-PFOS) and PFHxS (tot-PFHxS) are reported as a sum of the linear (*L*-) and *br*anched (*br*-) isomers.

The PFAS concentrations in fish are summarized in Table 3, while Table S3 details the results in each sample.

### 3.1. Concentrations and Profiles of PFASs in Fish Muscle

**Figure 1:**
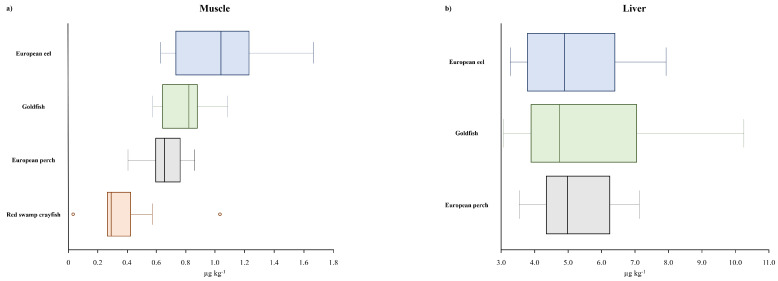
Box and whisker plot for Σ19PFASs (μg kg^−1^) in (**a**) muscle and (**b**) liver. Little circles represent outliers data.

Although the dataset obtained per species was small and the considered species numbered only four, the results were processed to obtain some statistical descriptors in order to try to highlight similarities and differences. Moreover, the results are presented using boxplots to better compare them using a graphical presentation. The concentrations of Σ19PFASs in the selected species ranged from 0.032 to 1.7 µg kg^−1^ (median 0.68 µg kg^−1^). The levels decreased in the order European eel > goldfish > European perch > red swamp crayfish. As shown in Figure 1a, among the species analyzed, European eels had the higher concentration variability, ranging from 0.63 to 1.7 µg kg^−1^, with a median value of 1.0 µg kg^−1^. In goldfish and European perch, median concentrations of 0.82 µg kg^−1^ (0.57–1.1 µg kg^−1^) and 0.65 µg kg^−1^ (0.41–0.86 µg kg^−1^) were quantified, respectively. Red swamp crayfish showed lower levels: Σ19PFASs ranged from 0.27 to 0.57 µg kg^−1^ (median: 0.29 µg kg^−1^), with two extreme values, 0.032 and 1.0 µg kg^−1^ (Figure 1a).

**Figure 2:**
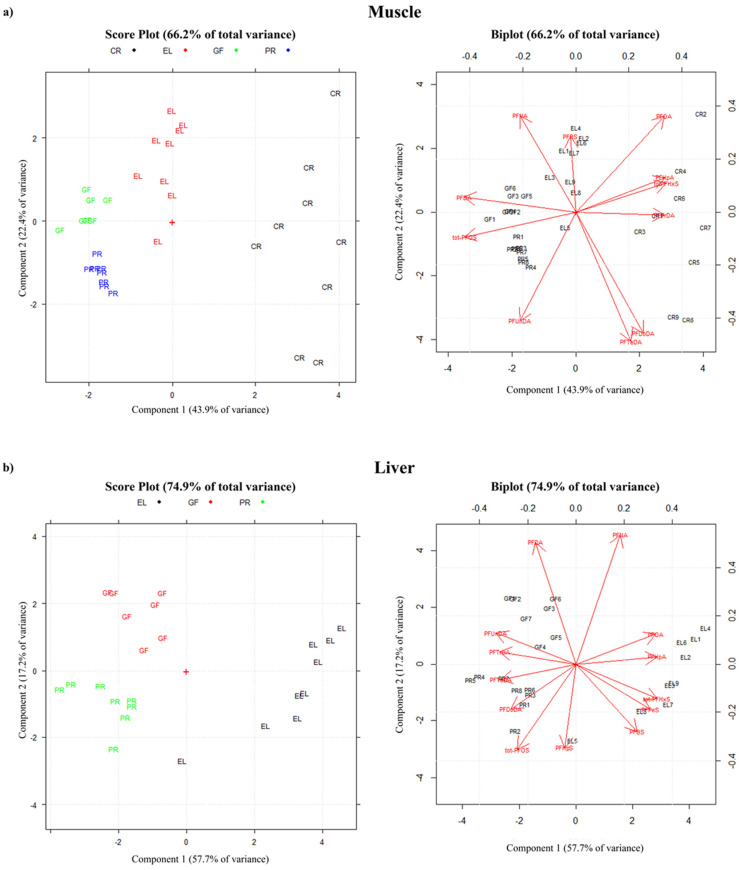
Principal component analysis (PCA) score plot (left) and loading plot (right) of PFAS contamination profiles in (**a**) muscle and (**b**) liver (GF: goldfish; PR: European perch; EL: European eel; CR: red swamp crayfish).

Although with the limited numbers obtained in the frame of the presented study, a very preliminary descriptive analysis of the contamination profile was carried out by PCA (Figure 2a). The PCA shows that PC1 and PC2 are able to explain 43.9% and 22.4% of the data variability, respectively (66.2 of the total variance). The loading plot indicates that in PC1, tot-PFOS, PFDA, and PFTrDA are the compounds with the highest impact, while in PC2, PFUnDA, PFTeDA, and PFDoDA have the greater influence.

The muscle samples group in four main areas on the plots: (1) European perches and (2) goldfish, which are very close groups, (3) European eels, and (4) red swamp crayfishes. The latter is located in a well-separated area, indicating differences in its contamination profile, with the main contributions being of the long-chain PFCAs (C8-C13) and of PFHxS.

**Figure 3:**
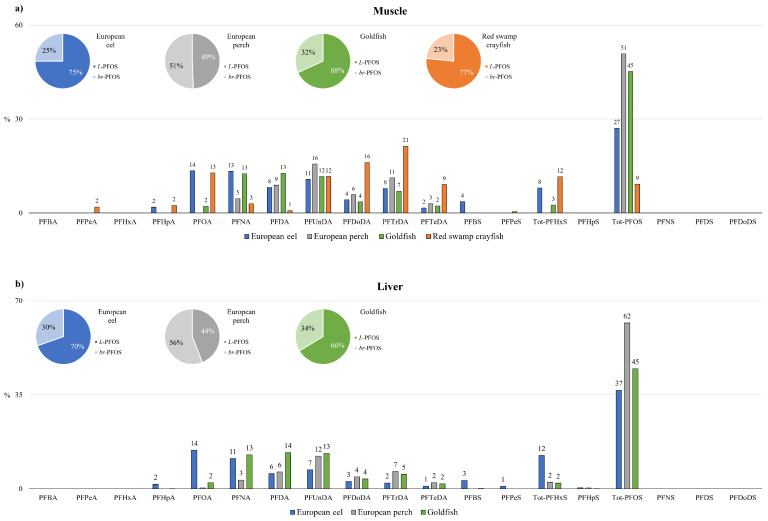
PFAS contamination patterns (%) in (**a**) muscle and (**b**) liver. The pie charts report branched (*br*) and linear (*L*) PFOSs’ contribution to total PFOS (%).

A detailed analysis of the contamination profiles is reported in Figure 3a. The PFAS profiles were quite similar among the fish species, but not in red swamp crayfish, suggesting the influence of species-specific accumulation, as reported in a previous study for other organic contaminants [32].

PFBA, PFHxA, PFHpS, PFNS, PFDS, and PFDoDS were below the quantification limits (LOQs) in all fishes and crustaceans analyzed. PFBS and PFHpA were quantified only in European eel’s muscle, while PFPeS was detected in only one goldfish sample. Traces of PFPeA were measured only in red swamp crayfish.

In fishes, tot-PFOS was the predominant compound, accounting, on average, for 41% of the total PFASs (between 27 and 51% of the Σ19PFASs). The long-chain PFCAs (C8-C14) provide a high contribution to the total PFAS contamination, with different patterns among species. In eel muscles, PFOA represented 14% of the contamination, followed by PFNA (13%) > PFUnDA (11%) > PFDA ≃ PFTrDA (8%). Only in European eels was PFHxS non-negligible, contributing, on average, 8% of the contamination, along with PFBS, which was always quantified. In European perch, PFOA was never measured, and the PFCAs C10-C14 were the most abundant (PFUnDA > PFTrDA > PFDA > PFDoDA), while in goldfish, the predominant PFCAs were C9-C11 in the order PFNA > PFDA > PFUnDA.

Unlike in the fishes, in the crustacean *P. clarkii*, tot-PFOS accounted for only 9% of the Σ19PFASs. The most contributing compounds in this species were the long-chain PFCAs: PFTrDA (21%), PFDoDA (16%), and PFUnDA (12%). PFOA was also relevant (13%).

The observation that PFOS and long-chain PFCAs in the fish samples were present in higher concentrations than shorter-chain compounds is consistent with findings from other studies [38,39]. However, long-chain PFCAs lack a specific dominant industrial or human activity source [29].

As regards the ratio between linear compounds and branched isomers, *br*-PFHxS was not measured in any of the muscle analyzed, while *br*-PFOS accounted for 23–32% to the tot-PFOS in European eels, goldfish, and red swamp crayfish, confirming in biota the preferential accumulation of linear isomers and/or the preferential excretion of branched isomers [40,41]. Only in the perch muscles was the level of the *br*anched isomers comparable with that of the linear PFOS (Figure 3a). Therefore, from the presented data, we can hypothesize that the ratio *L*/*br* in PFOS accumulation can differ among different species within the same ecosystem and may be influenced by factors such as the species’ metabolic pathway, diet, or habitat.

Although PCBs, heavy metals, organochlorine pesticides, and BFRs have been previously investigated, this study represents the first reporting on PFASs in fish from Lake Trasimeno, one of the most important Italian lakes [32,33,42]. Other studies on PFASs in freshwater fishes have been conducted, but very few have focused on the species selected in the present work. PFASs were never assessed in *P. clarkii*, while C. auratus was studied only in Chinese ecosystems [43,44].

Only *A. anguilla* and *P. fluviatilis* literature data are available; therefore, the obtained results were compared with those reported in the same species, collected in different Italian and European basins (Table S4).

The PFAS levels in the present samples were generally lower than those reported for fish collected in Northern Italian lakes and rivers. This was not unexpected, as Lake Trasimeno is an area characterized by low anthropogenic impact, while the Northern Italian regions are the most developed and industrialized. In accordance with our results, PFOS was the predominant compound in all the studies, followed by the long-chain PFCAs.

Regarding European perch, Squadrone et al. quantified PFOS in the muscle of specimens caught in Varese Lake, with a mean of 9.6 µg kg^−1^ (from 5.4 to 17 µg kg^−1^), while the PFOA level was <LOQ [28]. Valsecchi et al. reported PFAS concentrations in fishes from three different subalpine lakes (Varese, Lugano, and Mergozzo), located in Lombardy and Piedmont—regions with high anthropic impact [29]. Concentration ranges of 2.1–43 µg kg^−1^ for PFOS, 0.66–12 µg kg^−1^ for PFDA, 0.16–8.9 µg kg^−1^ for PFUnDA, and 0.015–4.8 µg kg^−1^ for PFDoDA were found in European perch. Mazzoni et al. reported PFAS levels in fishes from Mergozzo Lake; in this case, PFOS also had the highest levels (mean concentration 25 µg kg^−1^) [30]. Chiesa et al. investigated the PFAS concentrations in three Lombardy lakes (Garda, Como, Iseo); only PFOS was detected in perch samples (mean: 2.5 µg kg^−1^) [27]. The PFAS levels reported in the present study are much lower than those in samples from different European basins. Comparing only the PFOS concentration, mean values between 9.3 and 2.2 µg kg^−1^ were found in different Finnish lakes, while 10 and 8.0 µg kg^−1^ were measured in freshwater habitats in Flanders (Belgium) and Germany, respectively [20,21,23,45,46]. Instead, the PFAS level measured in perch from lakes in Sweden was comparable with that in European perch from Trasimeno, quantified at 0.44 and 0.13 µg kg^−1^ for PFOS, respectively [19,47].

In the case of European eels, the PFAS levels in specimens from Trasimeno were comparable with those reported in the same species from Po River and Garda Lake, where PFOS mean concentrations of 1.0 and 1.7 µg kg^−1^, respectively, were measured [24,26]. Higher PFAS levels were measured in different sampling sites located in the Loire estuary (France), where the PFOS level ranged between 11 and 25 µg kg^−1^, and in the Netherlands and Flanders, where 20 and 8.1 µg kg^−1^ of PFOS, respectively, were measured [22,46,48].

Unlike in the fishes, in *P. clarkii*, PFOS was measured at lower concentrations compared to long-chain carboxylic acids. The singular contamination pattern of this species was already reported for hexabromocyclododecanes (HBCD) [32]. It can be hypothesized that this is a result of a different crayfish metabolic pathway in the case of PFASs as well. Regarding PFASs, no data have been published on *P. clarkii*, but a previous study in gammarids, a family of marine amphipod crustaceans, showed that depuration was almost complete in 21 days for PFOS, but not for the long-chain PFCAs. Their depuration time increases as the chain length increases [46]. It could be interesting to investigate the accumulation of PFOS and long-chain PFCAs in red swamp crayfish, which is considered an optimal sentinel in ecotoxicological studies [47]. Long-chain PFCAs were also the predominant compounds in crabs (Hyas araneus) from the Norwegian Arctic, where PFCAs C8–C14 contributed 28% of the sum, while PFOS accounted for only 15% [41]. On the other hand, in shrimps and crustaceans collected in different coastal environments in Norway, PFOS was prevalently detected [48,49].

### 3.2. Concentrations and Profiles of PFASs in Fish Livers

The livers of *A. anguilla*, *P. fluviatilis*, and *C. auratus* were analyzed. In the case of livers, the dataset was limited, and the results were statistically analyzed to highlight similarities and differences. The results are presented in boxplots to provide a graphical comparison. The levels of PFASs in livers were generally significantly higher than those in muscles (*t*-test) (Table 2), confirming that perfluoroalkyl substances have a greater affinity for hepatic proteins and phospholipids [50,51,52,53].

The concentration of Σ19PFASs among the samples ranged from 3.1 to 10 µg kg^−1^, with a median of 4.8 µg kg^−1^.

While in the muscle, higher PFAS values were measured in eels, the concentrations in the livers of the selected species were rather comparable (Figure 1b). Median values of 4.9 µg kg^−1^, 5.0 µg kg^−1^, and 4.7 µg kg^−1^ were measured in European eel, European perch, and goldfish, respectively. Goldfish is, however, the species wherein the Σ19PFAS concentration was more variable, ranging between 3.1 and 10 µg kg^−1^. In European eels, the levels ranged between 3.3 and 7.9 µg kg^−1^, and in European perches, they ranged from 3.6 to 7.1 µg kg^−1^.

Short-chain PFCAs (PFBA, PFPeA, PFHxA) and the sulphonic acids PFNS, PFDS, and PFDoDS were not quantified in any of the livers analyzed. Among all the species, European eel had the highest number of compounds above the LOQs: the PFCAs C8-C14 and the PFSAs C4-C8 were always measured. Short-chain PFSAs (PFBS and PFPeS) were instead measured in only two samples of goldfish liver and never in European perch.

As already described for the muscles, a preliminary descriptive analysis of the contamination profile was performed by PCA (Figure 2b). The results show that PC1 and PC2 explain 57.7% and 17.2% of the data variation, respectively (74.9% of total variance). In the loading plot, PFUnDA, PFTrDA, PFOA, and PFHpA are the compounds with the highest impact on PC1, while PFDA, PFNA, and PFHpS have the highest impact on PC2. In the case of livers, only fish species were included in the analysis. The experimental data group into three distinct areas for (1) European perches, (2) goldfishes, and (3) European eels. The European eels are in a clearly separated cluster (Figure 2b), indicating a different profile.

A detailed liver contamination profile is reported in Figure 3b. Unlike what was observed in mammalian species, the PFAS profile in fish liver was quite similar to that in muscles: tot-PFOS was the predominant compound in all the species, contributing, on average, 48% of the total contamination [54]. In European eel livers, the profile perfectly fit with that for muscle: PFOA (14%) > PFHxS (12%) > PFNA (11%) > PFUnDA (7%) > PFDA (6%). In both goldfish and European perch, the contribution of the long-chain PFCAs C9-C11 (PFUnDA > PFDA > PFNA) was rather predominant (Figure 3b).

Unlike in muscle, *br*-PFHxS was measured in all the European eel livers, with a mean % ratio of 86/14 (*L-*/*br-*). In European eels and goldfish, *L*-PFOS was confirmed to be predominant with respect to the branched isomers, which accounted for 30% and 34% of the total PFOS, respectively. As in muscle, comparable levels of *br*- and *L*-PFOS (56% and 44%, respectively) were measured in European perch liver, confirming a species-specific accumulation profile (Figure 3b).

The fish species analyzed have the following trophic levels: European perch (4.4) < European eel (3.6) < goldfish (2.0) [55]. Differently from what was previously reported, no direct correlation was found between PFAS concentrations and the trophic levels of the Trasimeno fishes [56,57]. Goldfish, despite occupying a lower trophic level, had PFAS concentrations similar to those in the other species. This could potentially be explained by the specific habits of this benthic species feeding mainly on benthic invertebrates, which were found to have a strong ability to accumulate PFASs [58]. These findings suggest that the dynamics of PFAS accumulation in Lake Trasimeno’s ecosystem are complex. The unique feeding habits and interactions within the ecosystem may play a significant role in the distribution of PFASs among different species, highlighting the need for a more comprehensive understanding of these dynamics.

To the best of our knowledge, very few studies have investigated the PFAS levels in tissues other than muscle [59,60]. Giari et al. studied PFASs (only PFOS and PFOA) in eels caught in the Po River (Comacchio Lagoon, Italy) and found that the PFOS levels in liver were <0.40–4.3 µg kg^−1^, comparable with the levels in the Trasimeno European eels. The PFOA level, on the other hand, was higher (<0.40–85 µg kg^−1^) [24]. Faxneld et al. reported the PFAS concentrations in the muscle and liver of European perch sampled in Sweden. The levels in the livers were significantly higher compared to those in Trasimeno European perches, probably because some of the Swedish lakes involved in the monitoring were specifically chosen for the presence of possible PFAS contamination sources [47].

Significantly higher levels in perch livers were found in specimens sampled in Tyrifjorden Lake, Norway, a location close to paper factories and fire stations [61].

The present paper is the first to report results on 19 PFASs in the livers of freshwater fishes from a central Italian lake located in a low-anthropized area. Even though liver tissue is not typically used for human consumption, it is an essential indicator of environmental contamination within the ecosystem inhabited by these fish species.

The extent of PFAS contamination in the Umbria region, where Lake Trasimeno is located, remains uncertain. This monitoring may add some information to a precedential study on wild boars conducted in the Apennines mountains of the same region, highlighting the presence of PFASs, particularly PFOS, accumulating in the livers of wild animals [54]. What exactly are the possible exposure sources for these wild species in a region characterized by low anthropic impact and limited industrial activity? An accurate inventory of the possible sources in hypothetically non-contaminated areas may also be recommended.

### 3.3. Compliance with Regulatory Limits

Fishes and fish products from Lake Trasimeno have been and continue to be a vital food source for local communities. The lake, with its almost pristine environment and the absence of significant industrial activities, gives the perception of the fish as being of high quality and safe for consumption. This enduring practice of small-scale traditional fishing not only holds cultural significance but also aligns with the trend of promoting locally sourced food products: “farm-to-table” [34].

The PFAS levels in the analyzed freshwater species, all suitable for consumption, were compared with the maximum levels set by European Regulation 2023/915 for the four PFASs of greatest toxicological interest (PFOS, PFOA, PFNA, PFHxS) and their sum (Σ4PFASs) [7,13]. Only muscle samples were considered. Maximum levels of 2.0, 0.20, 0.50, 0.20, and 2.0 μg kg^−1^ for PFOS, PFOA, PFNA, PFHxS, and Σ4PFASs, respectively, were set for goldfish, with corresponding levels of 3.0, 0.70, 1.0, 1.5, and 5.0 μg kg^−1^ for red swamp crayfish. Higher limits were set for eel and European perch: 35 (PFOS), 8.0 (PFOA and PFNA), 1.5 (PFHxS), and 45 μg kg^−1^ (Σ4PFASs). The analyzed samples were all well below the maximum limits.

**Figure A1:**
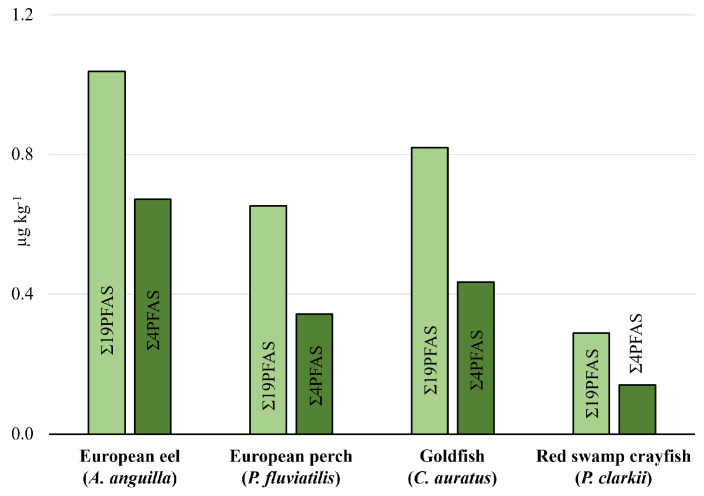
Σ19PFASs vs. Σ4PFASs (PFOS, PFOA, PFNA, PFHxS) in muscle (median values).

Figure A1 compares the average Σ19PFASs and Σ4PFASs in the muscle of each species. The sum of the four regulated compounds accounts for a substantial portion of the overall contamination—more than 55% of the total PFASs in fish and 48% in crayfish. However, the maximum limits do not account for an important portion of contamination, specifically, the long-chain carboxylic acids (C9-C14), for which no limits were issued but monitoring is still highly recommended under Commission Recommendation (EU) 2022/1431 [11].

### 3.4. Compliance with EQSs

Concerns about the undesired effects of pollutants in the aquatic environment are also acknowledged in Directive 2008/105/EC, which established Environmental Quality Standards (EQSs) for selected persistent organic contaminants (defined as priority hazardous substances) in surface water and biota, serving as thresholds below which no harmful effects are expected in wildlife and humans [16]. In the case of perfluoroalkyl substances, EQSs are currently set in biota only for PFOS at 9.1 μg kg^−1^. The values pertain to the whole organism; however, none of the samples analyzed in this study exceeded the EQS, whether considering the muscle or liver concentration, even though the liver is the primary target organ for PFOS accumulation, which is the main contributor to the total PFAS.

It is important to highlight that on the 26th of October 2022, the EU Commission released a draft amendment aiming to modify the three major water management Directives, including Directive 2008/105/EC [16,62,63,64].

The commission draft includes an updated list of EQSs for 68 substances and substance groups. The PFAS group will be considered as a sum of 24 compounds, and the sum-EQS will be expressed as the PFOA equivalent [65].

Therefore, to effectively assess PFAS contamination in the Lake Trasimeno ecosystem, a new evaluation will be necessary, considering the updated EQS limits.

## 4. Conclusions

This study reports the results of the first monitoring of four different species (*C. carassius*, *P. fluviatilis*, *A. anguilla*, and *P. clarkii*) harvested from Lake Trasimeno, located in a rural area of Central Italy. A total of seventy-three specimens, pooled into twenty-four (livers) and thirty-three (muscles) laboratory samples, were analyzed to assess the levels of 19 PFASs. They were all commercially relevant species and, therefore, a potential source of exposure for the local population. Thus, although the dataset was small and the considered species numbered only four, the collected information can be utilized for preliminary environmental monitoring and food contamination assessment. The total PFAS levels in livers were confirmed to be significantly higher than those in muscle in fish, and while the concentration in muscle decreased in the order European eel > goldfish > European perch > red swamp crayfish, in the liver, the levels were rather comparable among the species. As regards the PFAS contamination pattern, tot-PFOS was the predominant compound both in muscle and in livers. The long-chain PFCAs (C8-C14) contributed to the total PFAS contamination differently, depending on the species and reflecting species-specific differences in PFAS uptake, metabolism, and elimination processes. The ratio of linear PFOS to branched isomers was also studied: L-PFOS was predominant in both the liver and muscle of European eel, goldfish, and red swamp crayfish, while comparable levels of *br*- and *L*-PFOS were measured in European perch tissue. All the samples were largely below the maximum limits set by European Regulation 2023/915, and none exceeded the EQS established by Directive 2008/105/EC. PFAS EQSs are currently set only for PFOS, but they will shortly be revised considering the sum of 24 substances. Therefore, the PFAS contamination in Lake Trasimeno ecosystem will need to be reassessed. Although the numbers of samples and species considered are rather limited, the present study is the first to analyze 19 PFASs, both in livers and in muscles, in fishes caught from a lake in Central Italy. These findings support the need for ongoing monitoring, environmental protection, and regulatory actions.

## Figures and Tables

**Table 1:** Analyzed samples.

Species	Common Name	Specimens (n)	Laboratory Samples (n)	
			Muscle	Liver
*Anguilla anguilla*	European eel	9	9	9
*Perca fluviatilis*	European perch	16	8	8
*Carassius auratus*	Goldfish	7	7	7
*Procambarus clarkii*	Red swamp crayfish	41	9 *	

* Entire tissue remaining after removal of rostrum and carapace.

**Table 2:** List of analytes and respective internal standards.

N	Name	Acronym	Labelled Compound (ISs)
1	Perfluoro-n-butanoic acid	PFBA	[^13^C_4_] PFBA
2	Perfluoro-n-pentanoic acid	PFPeA	[^13^C_5_] PFPeA
3	Perfluoro-n-hexanoic acid	PFHxA	[^13^C_5_] PFHxA
4	Perfluoro-n-heptanoic acid	PFHpA	[^13^C_4_] PFHpA
5	Perfluoro-n-octanoic acid	PFOA	[^13^C_8_] PFOA
6	Perfluoro-n-nonanoic acid	PFNA	[^13^C_9_] PFNA
7	Perfluoro-n-decanoic acid	PFDA	[^13^C_6_] PFDA
8	Perfluoro-n-undecanoic acid	PFUnDA	[^13^C_7_] PFUnDA
9	Perfluoro-n-dodecanoic acid	PFDoDA	[^13^C_2_] PFDoDA
10	Perfluoro-n-tridecanoic acid	PFTrDA	[^13^C_2_] PFDoDA
11	Perfluoro-n-tetranoic acid	PFTeDA	[^13^C_2_] PFTeDA
12	Perfluoro-1-butanesulfonic acid	PFBS	[^13^C_3_] L-PFBS
13	Perfluoro-1-pentanesulfonic acid	PFPeS	[^13^C_3_] L-PFHxS
14	Perfluoro-1-hexanesulfonic acid *(linear isomer)*	*L-*PFHxS	[^13^C_3_] L-PFHxS
	Perfluoro-1-hexanesulfonic acid *(branched isomers)*	*br-*PFHxS	[^13^C_3_] L-PFHxS
15	Perfluoro-1-heptanesulfonic acid	PFHpS	[^13^C_3_] L-PFHxS
16	Perfluoro-1-octanesulfonic acid *(linear isomer)*	*L-*PFOS	[^13^C_8_] L-PFOS
	Perfluoro-1-octanesulfonic acid *(branched isomers)*	*br-*PFOS	[^13^C_8_] L-PFOS
17	Perfluoro-1-nonanesulfonic acid	PFNS	[^13^C_8_] L-PFOS
18	Perfluoro-1-decanesulfonic acid	PFDS	[^13^C_6_] PFDA
19	Perfluoro-1-dodecanesulfonic acid	PFDoDS	[^13^C_2_] PFDoDA

**Table 3:** PFAS concentrations (µg kg^−1^), given as median (min–max), in muscle and liver of freshwater species from Lake Trasimeno, Italy (Limit of Quantification (LOQ) = 0.010 µg kg^−1^; PFBA = 0.20 µg kg^−1^).

	European Eel (n = 9) (*A. anguilla*)		European Perch (n = 8) (*P. fluviatilis*)		Goldfish (n = 7) (*C. auratus*)		Red Swamp Crayfish (n = 9) (*P. clarkii*)
	MUSCLE	LIVER	MUSCLE	LIVER	MUSCLE	LIVER	MUSCLE
**PFBA**	n.c.	n.c.	n.c.	n.c.	n.c.	n.c.	n.c.
**PFPeA**	n.c.	n.c.	n.c.	n.c.	n.c.	n.c.	0.010 (<LOQ-0.022)
**PFHxA**	n.c.	n.c.	n.c.	n.c.	n.c.	n.c.	n.c.
**PFHpA**	0.018 (0.010–0.034)	0.11 (0.018–0.16)	n.c.	n.c.	n.c.	<LOQ (<LOQ-0.027)	0.010 (<LOQ-0.017)
**PFOA**	0.088 (0.024–0.30)	0.67 (0.12–1.6)	n.c.	0.019 (0.012–0.026)	0.010 (<LOQ-0.046)	0.056 (0.028–0.38)	0.048 (0.014–0.089)
**PFNA**	0.088 (0.040–0.28)	0.53 (0.19–1.2)	0.030 (0.027–0.035)	0.16 (0.11–0.31)	0.089 (0.071–0.18)	0.53 (0.25–1.7)	0.014 (<LOQ-0.022)
**PFDA**	0.083 (0.050–0.13)	0.27 (0.17–0.48)	0.060 (0.044–0.073)	0.32 (0.24–0.43)	0.084 (0.066–0.15)	0.71 (0.29–1.4)	<LOQ (<LOQ-0.028)
**PFUnDA**	0.11 (0.085–0.15)	0.37 (0.26–0.55)	0.10 (0.064–0.14)	0.63 (0.42–0.81)	0.095 (0.057–0.12)	0.69 (0.30–1.3)	0.036 (<LOQ-0.12)
**PFDoDA**	0.043 (0.030–0.059)	0.14 (0.087–0.21)	0.037 (0.021–0.054)	0.24 (0.18–0.27)	0.026 (0.022–0.037)	0.20 (0.10–0.38)	0.026 (<LOQ-0.24)
**PFTrDA**	0.079 (0.055–0.11)	0.11 (0.068–0.17)	0.077 (0.050–0.090)	0.35 (0.20–0.42)	0.061 (0.030–0.087)	0.35 (0.16–0.43)	0.064 (0.018–0.20)
**PFTeDA**	0.016 (0.012–0.022)	0.054 (0.032–0.080)	0.019 (0.015–0.024)	0.12 (0.083–0.17)	0.019 (0.012–0.024)	0.097 (0.053–0.21)	0.013 (<LOQ-0.13)
**PFBS**	0.039 (0.022–0.066)	0.19 (0.086–0.22)	n.c.	n.c.	n.c.	<LOQ (<LOQ-0.022)	n.c.
**PFPeS**	n.c.	0.048 (0.032–0.073)	n.c.	n.c.	<LOQ (<LOQ-0.025)	n.c.	n.c.
**PFHxS**	0.077 (0.048–0.18)	0.53 (0.31–0.97)	n.c.	0.11 (0.049–0.18)	0.018 (0.010–0.033)	0.10 (0.054–0.29)	0.053 (<LOQ-0.068)
***br-*PFHxS**	n.c.	0.081 (0.055–0.16)	n.c.	<LOQ (<LOQ-0.023)	n.c.	n.c.	n.c.
**PFHpS**	n.c.	0.022 (<LOQ-0.035)	n.c.	0.018 (0.012–0.036)	n.c.	<LOQ (<LOQ-0.035)	n.c.
***L-*PFOS**	0.20 (0.14–0.30)	1.3 (0.96–2.1)	0.14 (0.086–0.29)	1.2 (0.79–2.4)	0.22 (0.14–0.34)	1.3 (1.0–3.2)	0.023 (<LOQ-0.069)
***br-*PFOS**	0.067 (0.028–0.14)	0.57 (0.29–1.0)	0.18 (0.094–0.22)	1.5 (1.2–2.9)	0.10 (0.075–0.18)	0.67 (0.50–1.7)	0.011 (<LOQ-0.022)
**PFNS**	n.c.	n.c.	n.c.	n.c.	n.c.	n.c.	n.c.
**PFDS**	n.c.	n.c.	n.c.	n.c.	n.c.	n.c.	n.c.
**PFDoDS**	n.c.	n.c.	n.c.	n.c.	n.c.	n.c.	n.c.
**Σ19PFASs**	**1.0 (0.63–1.7)**	**4.9 (3.3–7.9)**	**0.65 (0.41–0.86)**	**5.0 (3.6–7.1)**	**0.82 (0.57–1.1)**	**4.7 (3.1–10)**	0.29 (0.032–1.0) *
**Σ4PFASs**	**0.67 (0.29–1.2)**	**3.9 (2.2–6.2)**	**0.34 (0.21–0.53)**	**3.3 (2.4–5.1)**	**0.43 (0.30–0.70)**	**2.5 (2.1–6.6)**	0.14 (0.11–0.17) **

n.c. = not calculated, all samples below Limit of Quantification * two outlier values not included (0.032 and 1.0 µg kg^−1^) ** two outlier values not included (0.014 and 0.27 µg kg^−1^).

## Data Availability

Data are contained within the article or Supplementary Material.

## Supplementary Materials

The following supporting information can be downloaded at: https://www.mdpi.com/article/10.3390/toxics12030196/s1 Table S1. Biometric data; Table S2. LC–MS/MS method; Table S3. Single-sample PFAS concentrations (LOQ = 0.010 µg kg^−1^; PFBA: 0.20 µg kg^−1^); Table S4. Comparison of concentration medians (range, µg kg^−1^ ww) of most frequently detected PFASs in European eels (*A. anguilla*) and European perches (*P. fluviatilis*) from different European countries.
