# Supplementary material for: Exploring Perfluoroalkyl Substances (PFASs) in Aquatic Fauna of Lake Trasimeno (Italy): Insights from a Low-Anthropized Area
Source: Toxics. 2024 Mar 1;12(3):196. doi: 10.3390/toxics12030196 (PMC10974762; doi:10.3390/toxics12030196)
Supplement: Supplementary file 1 [file toxics-12-00196-s001.zip › toxics-2822120-supplementary.pdf]

Table S1. Biometric data

Species	Common name	Length (cm) (min – max)	Weight (g) (min – max)
Anguilla anguilla	Eel	32 – 56	56 – 436
Perca fluviatilis	European perch	15 – 18	45 – 98
Carassius auratus	Goldfish	25 – 34	301 – 632
Procambarus clarkii	Red swamp crayfish	8 – 14	10 – 42

Table S2. LC-MS/MS method.

	CHROMATOGRAPHIC CONDITION							
					CONFIRMATORY			
**T° Coloum manager**	40 °C							
**T° Sample manager**	22 °C							
**Injection volume**	10 µL							
**Column**	Luna Omega PS C18 1.6 µm, 100 x 2.1 mm				Raptor C18 2.7 µm, 100 x 2.1 mm			
**Mobile phase A**	ACN				MeOH			
**Mobile phase B**	CH_3COONH_4 2 mM				CH_3COONH_4 2 mM			
	**Time (min)**	**Flow (mL/min)**	**A (%)**	**B (%)**	**Time (min)**	**Flow (mL/min)**	**A (%)**	**B (%)**
	0.00	0.100	10	90	0.00	0.100	10	90
	0.50	0.100	10	90	0.50	0.100	10	90
	2.00	0.200	10	90	2.00	0.250	10	90
	14.00	0.200	60	40	14.00	0.250	60	40
	21.00	0.200	100	0	21.00	0.250	100	0
	22.00	0.200	100	0	22.00	0.250	100	0
	22.10	0.100	10	90	22.10	0.100	10	90
	27.00	0.100	10	90	27.00	0.100	10	90

		MS/MS CONDITION							
**Capillary voltage**		0.90 kV							
**Desolvation temperature**		500 °C							
**Desolvation gas flow:**		1000 L/hr							
**Cone gas flow:**		20 L/hr							
**Ionization mode**		ES-							
		**Target transition (Tg)**			**Qualifier transition (Q)**				
		**Precursor ion (m/z)**	**Product ion (m/z)**	**Collision Energy (V)**	**Precursor ion (m/z)**	**Product ion (m/z)**	**Collision Energy (V)**	**Cone voltage (V)**	**Dwell (sec)**
PFBA	$C_4HF_7O_2$	213	169	10	213	213	5	6	0.0010
PFPeA	$C_5HF_9O_2$	263	219	10	263	263	5	10	0.0010
L-PFBS	$CF_3(CF_2)_3SO_3K$	299	80	30	299	99	30	10	0.0010
PFHxA	$C_6HO_2F_{11}$	313	269	10	313	119	24	4	0.0010
L-PFPeS	$C_5F_{11}SO_3Na$	349	80	30	349	99	30	10	0.0010
PFHpA	$C_7HF_{13}O_2$	363	319	5	363	169	16	6	0.0010
L-PFHxS	$C_6F_{13}SO_3Na$	399	80	35	399	99	35	10	0.0010
PFOA	$C_8HF_{15}O_2$	413	369	10	413	169	18	18	0.0010
L-PFHpS	$C_7F_{15}SO_3Na$	449	80	45	449	99	45	10	0.0010
PFNA	$C_9HF_{17}O_2$	463	419	5	463	219	16	6	0.0010
L-PFOS	$C_8F_{17}SO_3Na$	499	80	45	499	99	45	10	0.0010
PFDA	$C_{10}HF_{19}O_2$	523	469	10	513	219	16	10	0.0010
L-PFNS	$C_9F_{19}SO_3Na$	549	80	50	549	99	50	10	0.0010
PFUdA	$C_{11}HO_2F_{21}$	563	519	15	563	269	18	5	0.0010
L-PFDS	$C_{10}F_{21}SO_3Na$	599	80	50	599	99	50	10	0.0010
PFDoA	$C_{12}HF_{23}O_2$	613	569	10	613	169	25	10	0.0010
PFTrDA	$C_{13}HO_2F_{25}$	663	619	10	663	169	25	10	0.0010
L-PFDoS	$C_{12}F_{25}SO_3Na$	699	80	60	699	99	60	10	0.0010
PFTeDA	$C_{14}HO_2F_{27}$	713	669	15	713	169	25	10	0.0010

Table S3. Single samples PFASs concentrations (LOQ = 0.010 μg kg^{-1}; PFBA: 0.20 μg kg^{-1}) - MUSCLE

Eel (*Anguilla anguilla*) (μg kg^{-1})

n	1	2	3	4	5	6	7	8	9
**PFBA**	nq	nq	nq	nq	nq	nq	nq	nq	nq
**PFPeA**	nq	nq	nq	nq	nq	nq	nq	nq	nq
**PFHxA**	nq	nq	nq	nq	nq	nq	nq	nq	nq
**PFHpA**	0.029	0.034	0.014	0.025	0.010	0.032	0.018	0.010	0.012
**PFOA**	0.187	0.253	0.055	0.259	0.024	0.301	0.088	0.037	0.067
**PFNA**	0.205	0.179	0.088	0.257	0.040	0.281	0.085	0.053	0.066
**PFDA**	0.121	0.091	0.083	0.104	0.063	0.133	0.064	0.050	0.056
**PFUnDA**	0.142	0.116	0.100	0.117	0.112	0.146	0.096	0.085	0.099
**PFDoDA**	0.047	0.047	0.040	0.040	0.051	0.059	0.030	0.039	0.043
**PFTrDA**	0.092	0.079	0.055	0.074	0.105	0.095	0.079	0.080	0.077
**PFTeDA**	0.022	0.016	0.015	0.012	0.019	0.021	0.013	0.016	0.018
**PFBS**	0.045	0.040	0.035	0.029	0.030	0.022	0.066	0.039	0.041
**PFPeS**	nq	nq	nq	nq	nq	nq	nq	nq	nq
**L-PFHxS**	0.077	0.072	0.078	0.096	0.050	0.177	0.103	0.048	0.051
**br-PFHxS**	nq	nq	nq	nq	nq	nq	nq	nq	nq
**PFHpS**	nq	nq	nq	nq	nq	nq	nq	nq	nq
**L-PFOS**	0.237	0.202	0.300	0.169	0.148	0.301	0.257	0.139	0.157
**br-PFOS**	0.067	0.086	0.095	0.049	0.028	0.097	0.140	0.032	0.047
**PFNS**	nq	nq	nq	nq	nq	nq	nq	nq	nq
**PFDS**	nq	nq	nq	nq	nq	nq	nq	nq	nq
**PFDoDS**	nq	nq	nq	nq	nq	nq	nq	nq	nq
**Σ19PFAS**	**1.272**	**1.216**	**0.958**	**1.229**	**0.680**	**1.667**	**1.038**	**0.628**	**0.733**
**Σ4PFAS**	**0.774**	**0.792**	**0.617**	**0.829**	**0.289**	**1.158**	**0.672**	**0.310**	**0.388**

European perch (*Perca fluviatilis*) (μg kg^{-1})

n	1	2	3	4	5	6	7	8
**PFBA**	nq	nq	nq	nq	nq	nq	nq	nq
**PFPeA**	nq	nq	nq	nq	nq	nq	nq	nq
**PFHxA**	nq	nq	nq	nq	nq	nq	nq	nq
**PFHpA**	nq	nq	nq	nq	nq	nq	nq	nq
**PFOA**	nq	nq	nq	nq	nq	nq	nq	nq
**PFNA**	0.027	0.029	0.030	0.028	0.029	0.032	0.030	0.035
**PFDA**	0.065	0.073	0.050	0.057	0.063	0.044	0.048	0.071
**PFUnDA**	0.103	0.138	0.091	0.098	0.109	0.064	0.102	0.120
**PFDoDA**	0.039	0.036	0.031	0.046	0.049	0.021	0.033	0.054
**PFTrDA**	0.077	0.077	0.067	0.090	0.076	0.050	0.076	0.079
**PFTeDA**	0.018	0.019	0.017	0.024	0.023	0.015	0.018	0.023
**PFBS**	nq	nq	nq	nq	nq	nq	nq	nq
**PFPeS**	nq	nq	nq	nq	nq	nq	nq	nq
**L-PFHxS**	nq	nq	nq	nq	nq	nq	nq	nq
**br-PFHxS**	nq	nq	nq	nq	nq	nq	nq	nq
**PFHpS**	nq	nq	nq	nq	nq	nq	nq	nq
**L-PFOS**	0.292	0.262	0.132	0.127	0.165	0.086	0.113	0.148
**br-PFOS**	0.213	0.224	0.178	0.121	0.173	0.094	0.200	0.155
**PFNS**	nq	nq	nq	nq	nq	nq	nq	nq
**PFDS**	nq	nq	nq	nq	nq	nq	nq	nq
**PFDoDS**	nq	nq	nq	nq	nq	nq	nq	nq
**Σ19PFAS**	**0.834**	**0.858**	**0.596**	**0.592**	**0.685**	**0.407**	**0.621**	**0.686**
**Σ4PFAS**	**0.531**	**0.515**	**0.340**	**0.277**	**0.367**	**0.213**	**0.343**	**0.338**

Goldfish (*Carassius auratus*) (μg kg^{-1})

	1	2	3	4	5	6	7
**PFBA**	nq	nq	nq	nq	nq	nq	nq
**PFPeA**	nq	nq	nq	nq	nq	nq	nq
**PFHxA**	nq	nq	nq	nq	nq	nq	nq
**PFHpA**	nq	nq	nq	nq	nq	nq	nq
**PFOA**	nq	nq	0.031	0.010	0.031	0.046	nq
**PFNA**	0.107	0.071	0.097	0.075	0.089	0.177	0.076
**PFDA**	0.145	0.078	0.078	0.094	0.066	0.142	0.099
**PFUnDA**	0.122	0.075	0.075	0.101	0.057	0.119	0.095
**PFDoDA**	0.037	0.023	0.024	0.026	0.022	0.035	0.030
**PFTrDA**	0.064	0.087	0.035	0.035	0.030	0.071	0.061
**PFTeDA**	0.017	0.013	0.012	0.019	0.024	0.020	0.019
**PFBS**	nq	nq	nq	nq	nq	nq	nq
**PFPeS**	nq	nq	nq	nq	0.025	nq	nq
**L-PFHxS**	0.010	0.010	0.016	0.033	0.022	0.030	0.018
**br-PFHxS**	nq	nq	nq	nq	nq	nq	nq
**PFHpS**	nq	nq	nq	nq	nq	nq	nq
**L-PFOS**	0.220	0.143	0.179	0.311	0.183	0.344	0.322
**br-PFOS**	0.097	0.075	0.085	0.177	0.104	0.101	0.156
**PFNS**	nq	nq	nq	nq	nq	nq	nq
**PFDS**	nq	nq	nq	nq	nq	nq	nq
**PFDoDS**	nq	nq	nq	nq	nq	nq	nq
**Σ19PFAS**	**0.819**	**0.574**	**0.632**	**0.882**	**0.652**	**1.084**	**0.876**
**Σ4PFAS**	**0.434**	**0.298**	**0.408**	**0.607**	**0.428**	**0.698**	**0.571**

Red swamp crayfish (*Procambarus clarkii*) (μg kg^{-1})

	1	2	3	4	5	6	7	8	9
**PFBA**	nq	nq	nq	nq	nq	nq	nq	nq	nq
**PFPeA**	0.012	nq	nq	0.014	0.010	nq	0.010	nq	0.022
**PFHxA**	nq	nq	nq	nq	nq	nq	nq	nq	nq
**PFHpA**	0.010	nq	nq	0.010	0.012	0.014	0.012	0.010	0.017
**PFOA**	0.048	0.014	0.044	0.044	0.065	0.053	0.036	0.054	0.089
**PFNA**	0.014	nq	0.015	0.010	0.010	0.014	nq	0.020	0.022
**PFDA**	nq	nq	nq	nq	nq	nq	nq	nq	0.028
**PFUnDA**	0.036	nq	0.038	0.023	0.051	0.035	0.029	0.071	0.123
**PFDoDA**	0.026	nq	0.023	0.020	0.060	0.026	0.027	0.141	0.240
**PFTrDA**	0.064	0.018	0.052	0.063	0.105	0.048	0.078	0.113	0.204
**PFTeDA**	0.012	nq	0.013	nq	0.038	0.010	0.015	0.067	0.127
**PFBS**	nq	nq	nq	nq	nq	nq	nq	nq	nq
**PFPeS**	nq	nq	nq	nq	nq	nq	nq	nq	nq
**L-PFHxS**	0.028	nq	0.043	0.053	0.053	0.056	0.054	0.050	0.068
**br-PFHxS**	nq	nq	nq	nq	nq	nq	nq	nq	nq
**PFHpS**	nq	nq	nq	nq	nq	nq	nq	nq	nq
**L-PFOS**	0.027	nq	0.023	0.019	0.017	0.028	0.016	0.047	0.069
**br-PFOS**	0.012	nq	0.014	0.011	nq	0.016	nq	nq	0.022
**PFNS**	nq	nq	nq	nq	nq	nq	nq	nq	nq
**PFDS**	nq	nq	nq	nq	nq	nq	nq	nq	nq
**PFDoDS**	nq	nq	nq	nq	nq	nq	nq	nq	nq
**Σ19PFAS**	**0.288**	**0.032**	**0.265**	**0.267**	**0.421**	**0.299**	**0.277**	**0.572**	**1.029**
**Σ4PFAS**	**0.129**	**0.014**	**0.139**	**0.137**	**0.145**	**0.167**	**0.106**	**0.170**	**0.269**

Table S3 *(follow)*. Single samples PFASs concentrations (LOQ = 0.010 µg kg^{-1}; PFBA: 0.20 µg kg^{-1}) - LIVER

	Eel (*Anguilla anguilla*)									European perch (*Perca fluviatilis*)							
**n**	**1**	**2**	**3**	**4**	**5**	**6**	**7**	**8**	**9**	**1**	**2**	**3**	**4**	**5**	**6**	**7**	**8**
	**µg kg^{-1}**									**µg kg^{-1}**							
**PFBA**	nq	nq	nq	nq	nq	nq	nq	nq	nq	nq	nq	nq	nq	nq	nq	nq	nq
**PFPeA**	nq	nq	nq	nq	nq	nq	nq	nq	nq	nq	nq	nq	nq	nq	nq	nq	nq
**PFHxA**	nq	nq	nq	nq	nq	nq	nq	nq	nq	nq	nq	nq	nq	nq	nq	nq	nq
**PFHpA**	0.156	0.153	0.112	0.106	0.018	0.128	0.054	0.029	0.087	nq	nq	nq	nq	nq	nq	nq	nq
**PFOA**	1.549	1.476	0.497	1.120	0.117	1.019	0.257	0.211	0.672	0.019	0.020	0.016	0.017	0.022	0.018	0.026	0.012
**PFNA**	1.210	0.952	0.529	0.833	0.185	0.892	0.219	0.238	0.371	0.113	0.135	0.174	0.167	0.146	0.309	0.205	0.121
**PFDA**	0.480	0.423	0.359	0.276	0.247	0.364	0.171	0.181	0.233	0.299	0.403	0.295	0.344	0.351	0.428	0.280	0.242
**PFUnDA**	0.509	0.546	0.374	0.293	0.407	0.372	0.263	0.304	0.341	0.534	0.809	0.573	0.648	0.729	0.776	0.604	0.422
**PFDoDA**	0.168	0.214	0.140	0.091	0.214	0.152	0.087	0.126	0.143	0.229	0.265	0.189	0.269	0.266	0.258	0.209	0.175
**PFTrDA**	0.152	0.168	0.081	0.068	0.174	0.113	0.087	0.105	0.114	0.302	0.375	0.294	0.423	0.423	0.390	0.320	0.196
**PFTeDA**	0.064	0.075	0.054	0.032	0.080	0.053	0.038	0.049	0.057	0.106	0.130	0.103	0.126	0.170	0.137	0.113	0.083
**PFBS**	0.136	0.196	0.186	0.119	0.165	0.086	0.197	0.205	0.221	nq	nq	nq	nq	nq	nq	nq	nq
**PFPeS**	0.073	0.056	0.057	0.032	0.047	0.043	0.048	0.052	0.044	nq	nq	nq	nq	nq	nq	nq	nq
**L-PFHxS**	0.856	0.972	0.661	0.528	0.343	0.680	0.314	0.306	0.450	0.049	0.072	0.184	0.087	0.122	0.178	0.171	0.095
**br-PFHxS**	0.151	0.120	0.164	0.062	0.055	0.059	0.081	0.067	0.105	nq	nq	0.020	nq	0.016	0.014	0.023	nq
**PFHpS**	0.035	0.025	0.023	0.021	0.034	0.022	0.004	0.005	0.021	0.022	0.036	0.020	0.016	0.012	0.032	0.013	0.013
**L-PFOS**	1.565	1.620	2.139	0.959	1.396	1.347	1.068	1.035	1.144	2.246	2.454	1.233	1.123	0.999	1.690	0.854	0.795
**br-PFOS**	0.825	0.843	1.002	0.357	0.294	0.567	0.656	0.359	0.471	1.649	2.206	2.243	1.203	1.369	2.914	1.448	1.395
**PFNS**	nq	nq	nq	nq	nq	nq	nq	nq	nq	nq	nq	nq	nq	nq	nq	nq	nq
**PFDS**	nq	nq	nq	nq	nq	nq	nq	nq	nq	nq	nq	nq	nq	nq	nq	nq	nq
**PFDoDS**	nq	nq	nq	nq	nq	nq	nq	nq	nq	nq	nq	nq	nq	nq	nq	nq	nq
**Σ19PFAS**	**7.928**	**7.838**	**6.377**	**4.897**	**3.776**	**5.897**	**3.543**	**3.273**	**4.474**	**5.567**	**6.906**	**5.342**	**4.421**	**4.626**	**7.144**	**4.268**	**3.550**
**Σ4PFAS**	**6.156**	**5.982**	**4.991**	**3.859**	**2.391**	**4.564**	**2.595**	**2.216**	**3.213**	**4.076**	**4.888**	**3.870**	**2.596**	**2.674**	**5.122**	**2.726**	**2.419**

	Goldfish (*Carassius auratus*)						
	**1**	**2**	**3**	**4**	**5**	**6**	**7**
	**µg kg^{-1}**						
**PFBA**	nq	nq	nq	nq	nq	nq	nq
**PFPeA**	nq	nq	nq	nq	nq	nq	nq
**PFHxA**	nq	nq	nq	nq	nq	nq	nq
**PFHpA**	nq	0.027	nq	nq	nq	nq	nq
**PFOA**	0.039	0.056	0.225	0.028	0.142	0.385	0.036
**PFNA**	0.509	0.534	0.781	0.253	0.427	1.718	0.850
**PFDA**	0.788	0.706	0.684	0.331	0.292	1.396	1.216
**PFUnDA**	0.690	0.698	0.684	0.352	0.299	1.264	1.292
**PFDoDA**	0.204	0.240	0.176	0.100	0.108	0.379	0.265
**PFTrDA**	0.429	0.414	0.213	0.161	0.189	0.349	0.433
**PFTeDA**	0.097	0.104	0.091	0.062	0.053	0.215	0.133
**PFBS**	0.015	nq	nq	nq	nq	0.022	0.018
**PFPeS**	nq	nq	nq	nq	nq	nq	nq
**L-PFHxS**	0.059	0.054	0.123	0.096	0.104	0.289	0.118
**br-PFHxS**	nq	nq	nq	nq	nq	nq	nq
**PFHpS**	nq	nq	nq	nq	nq	nq	0.035
**L-PFOS**	1.335	1.152	1.515	1.048	1.058	3.159	2.577
**br-PFOS**	0.550	0.498	0.911	0.638	0.669	1.088	1.669
**PFNS**	nq	nq	nq	nq	nq	nq	nq
**PFDS**	nq	nq	nq	nq	nq	nq	nq
**PFDoDS**	nq	nq	nq	nq	nq	nq	nq
**Σ19PFAS**	**4.716**	**4.483**	**5.404**	**3.069**	**3.340**	**10.264**	**8.642**
**Σ4PFAS**	**2.493**	**2.294**	**3.555**	**2.063**	**2.399**	**6.638**	**5.250**

Table S4. Comparison of concentrations medians (range, µg kg^{-1} ww) of most frequently detected PFASs in eels (*Anguilla anguilla*) and European perches (*Perca fluviatilis*) from different European countries.

REFERENCE	SPECIES	SAMPLING	TISSUE	n	PFOA	PFNA	PFDA	PFUnDA	PFDoDA	PFTrDA	PFTeDA	PFHxS	PFOS
[22]		Italy (Po River, Comacchio Lagoon) January 2012 - June 2012		35	<0.40 (<0.40 - 24.7)								1.0 (<0.40 - 2.5)
[24]		Italy (Lake Garda)		90	0.18 (<0.010 - 0.54)	0.29 (<0.012 - 1.5)	0.58 (<0.030 - 4.4)	<0.035 (<0.035 - 1.8)	0.020 (<0.039 - 5.4)	<0.30 (<0.025 - 1.5)	<0.30 (<0.013 - 10.1)	<0.025 (<0.025)	1.7 (<0.0080 - 7.8)
[45]	Eel (*A. anguilla*)	Netherlands (different rivers, canals and lakes) 2010 - 2016	Muscle	86	<0.30 (<0.30 - 0.90)	<0.30 (<0.30 - 26.5)	5.0 (<0.30 - 15.7)	4.0 (<0.30 - 50.1)	3.1 (<0.30 - 17.7)	2.3 (<0.30 - 18.9)	1.4 (<0.30 - 12.1)	<0.30 (<0.30 - 2.0)	20.3 (3.3 - 67.2)
[43]		Belgium (Flanders) 2015 - 2018		41	0.29 (<0.11 - 0.73)	<0.49 (<0.49 - 0.61)	1.1 (<0.82 - 2.5)	0.85 (<0.45 - 7.2)	2.0 (<0.081 - 8.3)	0.86 (<0.13 - 6.6)	0.91 (<0.017 - 8.3)	<7.2 (<7.2)	8.1 (1.5 - 64.6)
**This study**		**Italy (Lake Trasimeno) March 2021**		**9**	**0.088 (0.024 - 0.30)**	**0.088 (0.040 - 0.28)**	**0.083 (0.050 - 0.13)**	**0.11 (0.085 - 0.15)**	**0.043 (0.030 - 0.059)**	**0.079 (0.055 - 0.11)**	**0.016 (0.012 - 0.022)**	**0.077 (0.048 - 0.18)**	**0.29 (0.17 - 0.40)**
[22]	Eel (*A. anguilla*)	Italy (Po River, Comacchio Lagoon) January 2012 - June 2012	Liver	35	<0.40 (<0.40 - 84.6)								1.7 (<0.40 - 4.3)
**This study**		**Italy (Lake Trasimeno) March 2021**		**9**	**0.67 (0.12 - 1.6)**	**0.53 (0.19 - 1.2)**	**0.28 (0.17 - 0.48)**	**0.37 (0.55 - 3.4)**	**0.14 (0.087 - 0.21)**	**0.11 (0.068 - 0.17)**	**0.054 (0.032 - 0.080)**	**0.59 (0.37 - 1.1)**	**1.7 (1.3 - 3.1)**

[a] Median range for different sample groups

[b] Mean (µg kg^{-1})

[c] Proximity to sites with suspected contamination

Table S4. *Follow*

REFERENCE	SPECIES	SAMPLING	TISSUE	n	PFOA	PFNA	PFDA	PFUnDA	PFDoDA	PFTrDA	PFTeDA	PFHxS	PFOS
[44]		Sweden (different lakes)<br>2011		17		0.040<br>(<0.020 - 0.10)	0.19<br>(0.090 - 0.46)	0.39<br>(0.20 - 1.3)	0.20<br>(<0.10 - 0.88)	0.63<br>(0.070 - 1.8)	0.080<br>(<0.050 - 0.39)		0.44<br>(0.13 - 3.8)
[26]		Italy (Lake Varese)<br>Autumn 2014		10	<LOQ<br>(<LOQ)								8.1<br>(5.4 - 17.2)
[17]		Sweden (different lakes)<br>2010 - 2011		80			0.065<br>(<0.0050 - 0.18)	0.24<br>(0.038 - 0.64)	0.11<br>(0.014 - 0.51)	0.23<br>(0.048 - 0.85)	0.022<br>(<0.013 - 0.11)		0.13<br>(<0.025 - 0.93)
[18]		Finland (different sites)<br>2014 - 2016		48	0.030[b]<br>(<LOQ - 0.060)	0.23[b]<br>(N.A. - 1.1)	0.50[b]<br>(N.A. - 1.6)	1.0[b]<br>(N.A. - 8.8)	0.23[b]<br>(N.A. - 0.52)	0.45[b]<br>(N.A. - 1.8)	0.090[b]<br>(<LOQ - 0.15)	0.030[b]<br>(<LOQ - 0.10)	3.4[b]<br>(N.A. - 18.0)
[28]		Italy (Lake Mergozzo)<br>November 2016		2	<LOD<br>(<LOD)	0.12<br>(<LOD - 0.12)	5.2<br>(2.5 - 7.9)	5.8<br>(2.7 - 8.9)	3.1<br>(1.4 - 4.8)			<LOD<br>(<LOD)	25.5<br>(13.0 - 38.0)
[43]	European perch (*P. fluviatilis*)	Belgium (Flanders)<br>2015 - 2018	Muscle	33	0.18<br>(<0.11 - 0.79)	<0.49<br>(<0.49 - 0.73)	1.6<br>(<0.82 - 4.6)	0.97<br>(<0.45 - 10.6)	1.1<br>(<0.081 - 5.4)	0.36<br>(<0.13 - 4.7)	0.29<br>(<0.017 - 4.3)	<7.2<br>(<7.2)	10.4<br>(2.7 - 53.5)
[27]		Italy (Lake Lugano, Lake Mergozzo, Lake Varese)<br>2015 - 2019		17	<LOD<br>(<LOD)	<LOD<br>(<LOD - 0.51)	5.2<br>(0.66 - 12.0)	1.4<br>(0.16 - 8.9)	1.5<br>(0.015 - 4.8)	<LOD<br>(<LOD - 1.2)	<LOD<br>(<LOD - 0.44)	<LOD<br>(<LOD)	12.9<br>(2.1 - 42.6)
[19]		Finland (different sites)<br>2016 - 2017		10	<0.33<br>(<0.33)	0.37<br>(<0.25 - 0.82)	0.28<br>(<0.28 - 0.49)	0.48<br>(<0.32 - 0.80)	<0.32<br>(<0.32)	0.37<br>(<0.36 - 0.52)			2.2<br>(1.3 - 4.7)
[42]		Finland (firefighting training sites)[c]<br>2016 - 2017		5	0.11[b]<br>(<0.010 - 0.026)	0.39[b]<br>(0.093 - 0.68)	0.59[b]<br>(0.35 - 1.6)	0.73[b]<br>(0.53 - 1.4)	0.16[b]<br>(0.068 - 0.23)	0.29[b]<br>(0.19 - 0.50)	0.048[b]<br>(<0.040 - 0.055)	0.068[b]<br>(<0.010 - 0.11)	9.3[b]<br>(5.3 - 22.4)
[21]		Germany (different rivers, lakes, lagoons)<br>2016 - 2017		11	0.030[b]<br>(0.020 - 0.060)	0.12[b]<br>(0.030 - 0.23)	2.0[b]<br>(0.43 - 4.6)	0.98[b]<br>(0.20 - 1.5)	1.7[b]<br>(0.050 - 3.4)	0.91[b]<br>(0.080 - 1.4)	0.84[b]<br>(0.020 - 1.9)		8.0[b]<br>(1.5 - 16.0)
[25]		Italy (Lake Garda, Lake Iseo, Lake Como)		36	<0.050<br>(<0.050)	<0.050<br>(<0.050)	<0.050<br>(<0.050)	<0.050<br>(<0.050)	<0.050<br>(<0.050)	<0.10<br>(<0.10)	<0.10<br>(<0.10)	<0.050<br>(<0.050)	2.5[b]<br>(<0.050 - 4.4)
**This study**		**Italy (Lake Trasimeno)<br>January 2021**		**8**	**<0.010<br>(<0.010)**	**0.030<br>(0.027 - 0.035)**	**0.060<br>(0.044 - 0.073)**	**0.10<br>(0.064 - 0.14)**	**0.037<br>(0.021 - 0.054)**	**0.077<br>(0.050 - 0.090)**	**0.019<br>(0.015 - 0.024)**	**<0.010<br>(<0.010)**	**0.31<br>(0.18 - 0.51)**
[44]		Sweden (different lakes)<br>2011		17		0.41<br>(0.17 - 0.90)	2.5<br>(1.2 - 8.7)	5.6<br>(1.0 - 16.0)	2.9<br>(0.58 - 10.0)	4.5<br>(0.63 - 14.0)	0.99<br>(0.19 - 3.1)		8.1<br>(3.1 - 126)
[61]	European perch (*P. fluviatilis*)	Norway (Lake Tyrifjorden)[c]<br>June 2018 - October 2018	Liver	27	0.50<br>(<0.30 - 7.3)	0.40<br>(<0.30 - 3.6)	19.5<br>(9.3 - 29.0)	14.0<br>(10.0 - 47.0)	22.5<br>(9.4 - 78.0)	13.0<br>(6.3 - 54.0)	8.9<br>(1.7 - 27.0)		159<br>(85.0 - 371)
**This study**		**Italy (Lake Trasimeno)<br>January 2021**		**8**	**0.019<br>(0.012 - 0.026)**	**0.165<br>(0.11 - 0.31)**	**0.32<br>(0.24 - 0.43)**	**0.63<br>(0.42 - 0.81)**	**0.24<br>(0.18 - 0.27)**	**0.35<br>(0.20 - 0.42)**	**0.12<br>(0.083 - 0.17)**	**0.12<br>(0.049 - 0.20)**	**2.9<br>(2.2 - 4.7)**

[a] Median range for different sample groups
[b] Mean (μg kg^{-1})
[c] Proximity to sites with suspected contamination
